# Guide to Therapeutics and Materia Medica

**Published:** 1889-06

**Authors:** 


Guide to Therapeutics and Materia Medica. By Roburt Farquharson, M. P., M. D., Edin., F. R. C. P., Lond., L., L. D., Aber., pp. 598. Issued by Lea Bros. & Co., Philadelphia, 1889.

We have before us this neatly bound and well arranged book which shows great study and thought by its author.

It is somewhat differently arranged from the common run of such works. After a fair inspection and perusal of its contents, we have no hesitation in heartily commending it to the great body of men who are seeking scientific knowledge in this special line.

